# Study on the Synergistic Enhancement of Mechanical Properties of Magnesia–Chrome Refractory Bricks Through Component Ratio Optimization and Salt Impregnation Process

**DOI:** 10.3390/ma19091878

**Published:** 2026-05-02

**Authors:** Liming Zou, Yuefeng Qi, Benjun Cheng, Wencheng Wang, Kuiqing Guo

**Affiliations:** 1 School of Energy Science and Engineering, Central South University, Changsha 410083, China; 253911025@csu.edu.cn (L.Z.); 253912062@csu.edu.cn (W.W.); 253912037@csu.edu.cn (K.G.); 2 Henan Zhongyuan Special Steel Equipment Manufacturing Co., Ltd., Jiyuan 419001, China; 15639136210@163.com

**Keywords:** magnesia–chrome bricks, synergistic strengthening, component ratio, vacuum salt impregnation, mechanical properties, sintering performance

## Abstract

To meet the stringent industrial service requirements of magnesia–chrome refractory bricks, this study adopts a technical approach that synergistically combines precise component ratio optimization with a vacuum-pressure MgSO_4_ salt impregnation process to investigate the performance optimization of magnesia–chrome bricks. Samples were prepared by controlled formulation mixing, pressing at 250 MPa, drying at 110 °C, and firing at 1750 °C. Phase composition, microstructure, and physical–mechanical properties were characterized by XRD, SEM, and standard refractory test methods. The optimal additions of chromite powder and Cr_2_O_3_ micro-powder were determined to be 3 wt.% and 2 wt.%, respectively, which reacted with periclase to form a secondary composite spinel, creating a dense spinel bridge network that connected adjacent grains. Furthermore, when the proportion of sintered magnesia powder (MgO > 97 wt.%) was increased to 11 wt.%, the material achieved efficient densification facilitated by enhancing sintering performance. Based on this optimized formulation, and due to the high elemental compatibility between MgSO_4_ and the magnesia–chrome brick matrix as well as the excellent permeability of the solution, the MgSO_4_ vacuum-pressure salt impregnation process was subsequently applied. The salt solution filled the open pores and microcracks of the material, forming a crystalline salt micro-pillar reinforcing phase. Consequently, the apparent porosity of the material decreased to 10.98%, the bulk density increased to 3.23 g/cm^3^, and the cold compressive strength and cold modulus of rupture reached as high as 113.52 MPa and 24.91 MPa, respectively. This study innovatively establishes a new pathway for enhancing the mechanical properties of magnesia–chrome refractory bricks through the synergistic design of component ratio optimization and salt impregnation process. The prepared magnesia–chrome refractory bricks exhibit both excellent mechanical properties and volume stability.

## 1. Introduction

Magnesia–chrome bricks, primarily composed of magnesia (MgO) and chromic oxide (Cr_2_O_3_), are manufactured from magnesia-based raw materials (such as fused magnesia and sintered magnesia) and chrome-based raw materials (including chrome concentrate and industrial chromic oxide). These bricks exhibit excellent thermal stability, a low thermal expansion coefficient, and chemical inertness towards molten metals and slags, leading to their widespread application in metallurgical processes such as cement rotary kilns, copper converters, and secondary refining operations [1,2]. In equipment such as RH degassers and copper-smelting side-blown furnaces, the performance of magnesia–chrome refractory bricks directly dictates the equipment’s continuous operation cycle; fluctuations in their performance can lead to decreased production efficiency and a significant increase in maintenance costs and raw material consumption [3]. Current high-end metallurgical equipment imposes comprehensive performance requirements on magnesia–chrome bricks, such as high mechanical strength, low porosity, and excellent volume stability. Traditional magnesia–chrome refractory bricks are no longer able to meet the stringent service conditions of next-generation metallurgical equipment.

To enhance the comprehensive performance of magnesia–chrome bricks, numerous studies have been conducted by researchers globally, as detailed below: Guo et al. [4] found that the formation and content of secondary spinel are key to improving the high-temperature performance of magnesia–chrome bricks, with its content increasing as the firing temperature rises. Kim et al. [5] reported that chromite in MgO-Cr_2_O_3_ refractories can react with FeO to form high-melting-point composite spinel. This reactivity confers stability to the chromite, resulting in excellent resistance to FeO corrosion. Liu et al. [6] investigated the influence of atmosphere on magnesia–chrome refractories, revealing that a reducing atmosphere leads to the partial reduction of Fe^3+^/Fe^2+^ within the spinel to metallic Fe, which aggravates slag penetration and degrades material performance. The temperature-dependent variation of Young’s modulus is a characteristic property of magnesia–chrome refractories, exhibiting a hysteresis-like loop in its curve. As the Cr_2_O_3_ content in the material increases, the area of the hysteresis loop between the heating and cooling curves progressively diminishes [7,8]. The damage of magnesia–chrome refractory bricks is mainly manifested by slag penetration and corrosion, as well as structural spalling caused by thermal stress resulting from differences in thermal expansion [9]. The erosion channels for cement clinker liquid phase and alkali salts at high temperatures are the open pores of direct-bonded magnesia–chrome bricks. Reducing the open porosity of direct-bonded magnesia–chrome refractory bricks can effectively decrease their damage rate in the burning zone.

Regarding the preparation of magnesia–chrome bricks, numerous researchers have dedicated themselves to in-depth studies focusing on vacuum impregnation optimization, low-temperature sintering aids, and in situ spinel toughening. Deng et al. [10] employed Cr_2_O_3_ precursor sol and MgCr_2_O_4_ spinel precursor sol for vacuum impregnation treatment of magnesia–chrome refractories. The resulting materials exhibited superior properties in terms of bulk density, cold compressive strength, pore size distribution, and chemical composition compared to the untreated samples. The core principle of low-temperature sintering processes is to reduce the reaction temperature between MgO and Cr_2_O_3_ and promote the sintering process through additive modification or raw material activation treatments [11]. Introducing aluminum titanate into magnesia–chrome refractory bricks utilizes the TiO_2_ and Al_2_O_3_ generated from its high-temperature decomposition to form low-melting-point continuous phases with components such as CaO and SiO_2_ in the matrix, thereby enhancing the mass transfer process and improving thermal shock stability. Azhari et al. [12] found that the introduction of nano-iron oxide promoted the formation of magnesium–iron spinel at low temperatures. The dissolution of iron oxide and ion migration improved the sintering process of the refractory matrix. Furthermore, the presence of nano-iron oxide facilitated the formation of direct bonds while inhibiting silicate bonding. Hu et al. [13,14] found that ZrO_2_, acting as a sintering aid, helps to eliminate open pores and enhance interfacial bonding between grains in magnesia–chrome refractories, and induces a crack deflection effect, thereby improving the material’s modulus of rupture and fracture toughness. Meanwhile, vacuum impregnation with zirconia sol significantly increases the material’s bulk density, and the introduced zirconia particles enhance the material’s resistance to corrosion and penetration. An et al. [15], based on a system of fused magnesia–chrome particles and aluminum powder, achieved strengthening and toughening of a particle-stacked porous magnesia–chrome permeable material through an in situ spinel solid solution. This led to a reduction in the median pore size and an increase in the fractal dimension of the pore surfaces, resulting in a significant enhancement of the material’s mechanical properties.

To meet increasingly stringent service conditions, high-end magnesia–chrome brick products achieve excellent performance [2,16,17] through the use of high-purity raw materials, nano-additives, high-temperature firing, and precision processing. Against the backdrop of growing resource constraints and intensified market competition, the key to enhancing the core competitiveness of China’s refractory industry lies in reducing the manufacturing cost and simplifying the production process of magnesia–chrome refractory bricks without compromising, and even improving, their critical service performance. However, existing research on enhancing the performance of magnesia–chrome refractory bricks mostly focuses on single technical pathways, either emphasizing the optimization of raw material ratios and in situ spinel generation, or individually employing post-treatment methods such as sol impregnation and sintering aid addition. Meanwhile, mainstream impregnation processes mostly use chromium-based or zirconium-based precursor sols, which suffer from issues such as high raw material costs, complex preparation procedures, and limited compatibility with the magnesia–chrome matrix, making it difficult to balance economic viability and scalability for industrial production. This paper will conduct research focusing on the optimization of raw material ratios and the mechanism of vacuum salt impregnation for magnesia–chrome bricks, aiming to provide a theoretical foundation and technical support for the industrial production of high-performance magnesia–chrome bricks.

## 2. Experimental Preparation

### 2.1. Experimental Materials

The raw materials used in the experiment include chromite powder (325 mesh), Cr_2_O_3_ micro-powder (Cr_2_O_3_ > 99 wt.%, 5 μm), fused magnesia (1.5–3 mm, MgO > 98 wt.%), fused magnesia powder (200 mesh), fused magnesia–chrome (0.5–1 mm), and sintered magnesia powder (MgO > 97 wt.%, 200 mesh). The above raw materials all meet industrial-grade refractory production standards and were supplied by Henan Hexin Refractory Materials Co., Ltd. in Luoyang, Henan Province, China. Their main component contents are shown in Table 1.

### 2.2. Experimental Formulations

Based on the current component ratios of magnesia–chrome refractory bricks in actual production, the objective of this study is to establish a fundamental raw material system for magnesia–chrome refractory bricks with a reasonable particle size distribution, stable sintering behavior, and reproducible process. Fused magnesia was fixed at 30 wt.% as the main aggregate to provide a stable structural skeleton and high-temperature resistance. Meanwhile, fused magnesia–chrome (26 wt.%) and chromite (10 wt.%) were kept constant to synergistically optimize the particle size distribution. Domestic magnesia–chrome refractory bricks commonly use chromite, and rarely introduce highly active Cr_2_O_3_ micro-powder for precise regulation. To balance production cost and performance requirements, Cr_2_O_3_ micro-powder (0–3 wt.%) was introduced to investigate properties of magnesia–chrome refractory bricks. Controlled experiments were conducted by varying a single raw material variable, with the specific formulation compositions shown in Table 2.

For formulations 1–4, the content of Cr_2_O_3_ micro-powder was fixed at 0 wt.%, and the optimal ratio was investigated by gradually increasing the content of chromite powder. For formulations 5–8, the content of chromite powder was fixed at 3 wt.%, and the optimal ratio was investigated by gradually increasing the content of Cr_2_O_3_ micro-powder. Formulations 9–11 investigate the effects of different magnesia powder types on sintering densification and volume stability by setting a gradient ratio of sintered magnesia powder to fused magnesia powder based on a fixed mixture of “2 wt.% Cr_2_O_3_ micro-powder + 3 wt.% chromite powder.”

### 2.3. Experimental Methods

All raw materials were accurately weighed according to the formulations, and the fine powders were premixed uniformly for subsequent use. A pan mixer was adopted for mixing. Firstly, all the weighed granular materials were added into the pan mixer, and the equipment was turned on for dry mixing for 3 min to achieve initial homogeneity of the granules. An appropriate amount of pulp powder solution was then added for wet mixing for 5 min to ensure that the binder uniformly coated the surface of the granules. Subsequently, the pre-mixed fine powders were added, and wet mixing was continued for 7 min to ultimately obtain a homogeneously mixed batch.

The uniformly mixed batch was evenly loaded into a standard mold of size 25 mm × 25 mm × 150 mm, and pressed on a hydraulic press at a pressure of 250 MPa. The formed samples were then dried in an oven at 110 °C for 24 h. After drying, the samples were directly placed into a high-temperature furnace for firing. They were first heated to 900 °C at a rate of 5 °C/min, and then to 1750 °C at a rate of 3 °C/min, followed by a holding time of 3 h to obtain the final magnesia–chrome brick products. For each experimental formulation, three samples were prepared, and the above procedure was repeated twice.

**Figure 1:**
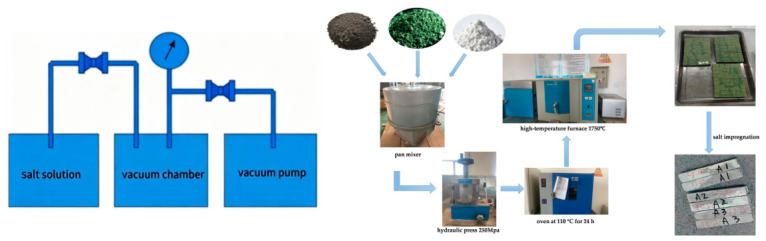
MgSO_4_ vacuum impregnation apparatus and experimental procedure.

The products of formulations 9–11 were then subjected to vacuum-pressure salt impregnation. First, MgSO_4_ powder was dissolved in water at 100 °C to prepare a saturated solution. The fired products were then placed into an impregnation tank. A vacuum pump was used to evacuate the tank to −0.1 MPa and held for 10 min to remove air from the pores within the products. Subsequently, the MgSO_4_ salt solution was introduced until it exceeded the top surface of the products with a sufficient volume for impregnation, and then the solution supply was stopped. The tank was evacuated again to −0.1 MPa using the vacuum pump and held for another 10 min. Then, compressed air was introduced into the tank to reach 0.8 MPa and held under pressure for 20 min to allow the salt solution to penetrate into the pores of the products to the greatest possible extent. Finally, the impregnated samples were removed and dried at 110 °C for 12 h to obtain the final salt-impregnated products. Figure 1 briefly shows the salt impregnation apparatus and the preparation process.

### 2.4. Performance Testing and Characterization

The bulk density and apparent porosity of the samples were tested according to GB/T 2997–2015 [18], using the water displacement method by measuring the dry mass and the suspended mass after saturation. The cold compressive strength of the samples was tested following GB/T 5072-2023 [19]. The cold modulus of rupture (CMOR) of the samples was tested according to GB/T 3001-2017 [20]; the three-point bending test was conducted at room temperature, in which bar-shaped specimens were loaded at a constant speed at the midspan until fracture. The hot modulus of rupture of the samples was tested following GB/T 3002-2017 [21]. For each formulation, three parallel samples were prepared, and the experiment was repeated twice. Thus, for each performance test, there were six experimental data points per formulation, and the average value was taken.

A Gemini SEM 300 field-emission scanning electron microscope (Zeiss, Germany) was used to observe the microstructure, grain morphology, pore filling state, and interfacial bonding characteristics of the samples, with simultaneous EDS analysis for elemental composition. Phase analysis was performed using a MiniFlex600 X-ray diffractometer (Rigaku, Kyoto, Japan) with a scanning speed of 10°/min and a scanning range of 2θ = 5–90° for phase identification. The phases were calibrated by matching with standard PDF cards. ImageJ 2024 software was used to measure and mark the diameters of the pores in the SEM images.

## 3. Results and Discussion

This study investigates magnesia–chrome refractory bricks through systematic adjustment of matrix composition and application of a vacuum-pressure salt impregnation post-treatment process. The measured average performance data are shown in Table 3, Table 4 and Table 5. The error bars in the line charts represent the standard deviation of multiple tests, and the results are expressed as mean ± standard deviation to reflect the dispersion of the experimental data and the repeatability of the tests.

### 3.1. Study on Component Ratio Optimization

#### 3.1.1. Study on the Addition Amount of Chromite Powder

**Figure 2:**
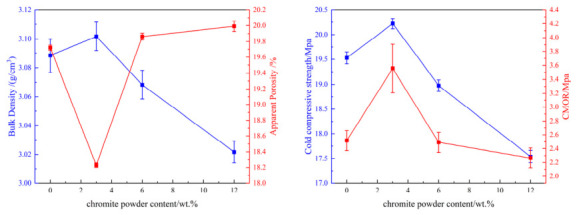
Effect of chromite powder content on performance.

Firstly, the effect of chromite powder addition was investigated, with specific formulations detailed in Table 2. The experimental results indicate that as the chromite powder content increases, the bulk density, cold compressive strength, and modulus of rupture of the material initially increase and then decrease, while the apparent porosity exhibits an opposite trend, as shown in Figure 2.

**Figure 3:**
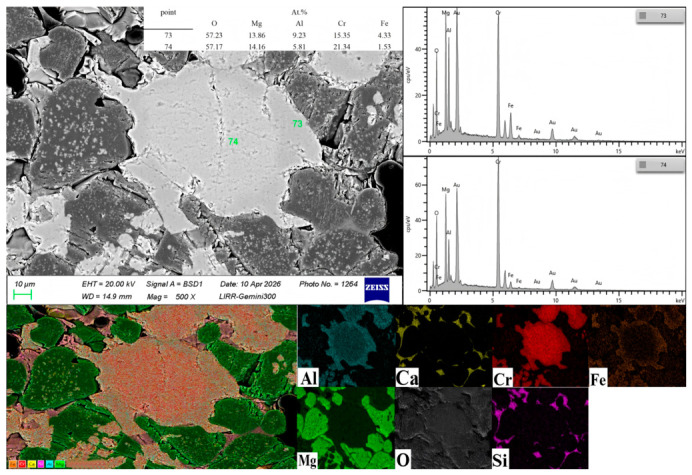
Microstructure image of the sample with 3 wt.% chromite powder.

As shown in Figure 3, the bright-white area in the middle is the fine chromite powder, with a particle size of approximately 0.1 mm, which is closely bonded to the magnesia matrix. Point 74, located inside the chromite fine powder, has a significantly higher Cr content than Point 73 (outside). The Cr element from the exterior of the chromite fine powder diffuses abundantly into the magnesia matrix, while the diffusion rate of Cr from the interior to the exterior is slower. The Cr element diffused from the chromite integrates into the magnesia material, subsequently precipitating fine-grained, well-dispersed magnesia–chrome spinel (MgCr_2_O_4_). In direct-bonded magnesia–chrome bricks, periclase and spinel grains form strong direct bonds with few low-melting phases, resulting in high hot strength, good corrosion resistance, and excellent thermal shock stability [16,22]. This results in increased bulk density, decreased apparent porosity, and enhanced cold compressive strength and modulus of rupture. Oxides such as Fe_2_O_3_ and Al_2_O_3_ diffused from the chromite powder form solid solutions within the magnesia–chrome spinel, thereby relatively reducing the negative impact of these impurities on the material’s high-temperature performance. However, an excessively high addition of chromite powder introduces too many low-melting-point impurities, leading to a significant increase in the glassy impurity phase formed during sintering [23].

#### 3.1.2. Study on the Addition Amount of Cr_2_O_3_ Micro-Powder

**Figure 4:**
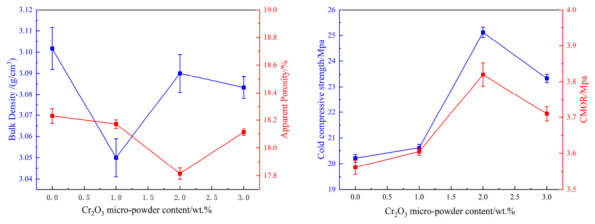
Effect of Cr_2_O_3_ micro-powder content on performance.

From the aforementioned study, it was determined that the addition of 3 wt.% chromite powder yields favorable sintering performance. To further enhance the material’s properties, we conducted research on introducing highly reactive Cr_2_O_3_ micro-powder. As can be seen from Figure 4, with the addition of Cr_2_O_3_ micro-powder, the apparent porosity first decreases and then increases, while the cold compressive strength and modulus of rupture initially increase and subsequently decrease.

**Figure 5:**
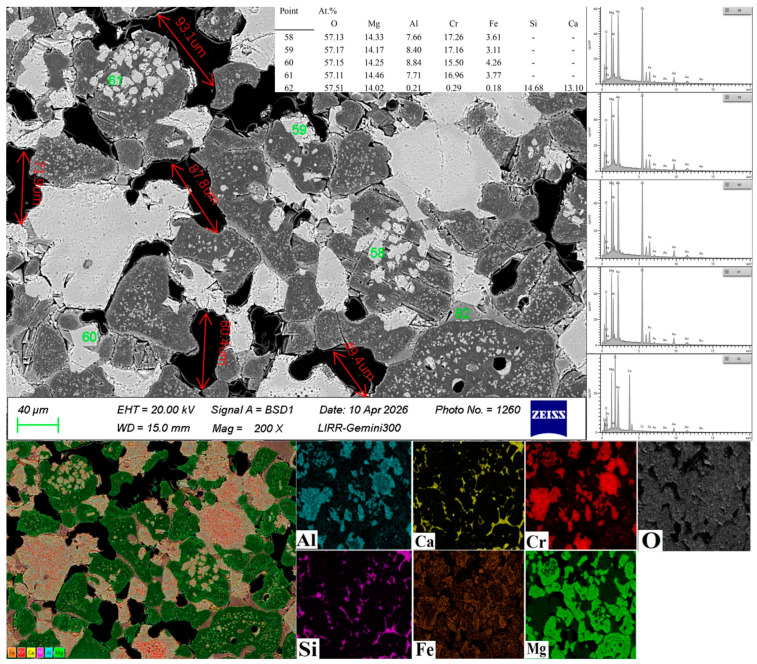
Microstructure image of the sample with 2 wt.% Cr_2_O_3_ micro-powder.

**Figure 6:**
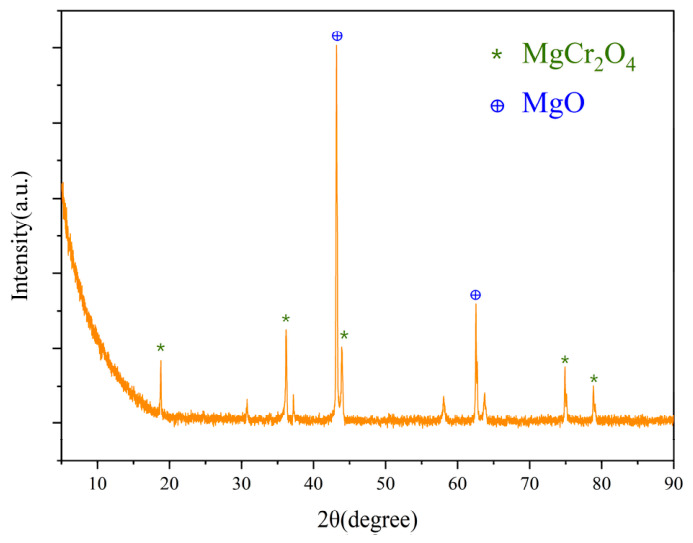
XRD of the sample with 2 wt.% Cr_2_O_3_ micro-powder.

From the analysis of Figure 5 and Figure 6, it can be concluded that Points 58 and 61 are magnesia–chrome spinel precipitated from the reaction between chromite and MgO; Points 59 and 60 are located at the grain-boundary gaps of the periclase aggregates, representing intergranular magnesia–chrome spinel phase. This phase bridges adjacent periclase grains, forming a continuous and interconnected spinel bridge network. Owing to its high specific surface area and enhanced chemical reactivity, Cr_2_O_3_ micro-powder readily reacts with MgO at relatively lower sintering temperatures, enabling the in situ generation of large amounts of high-purity secondary MgCr_2_O_4_ spinel. This fine-grained spinel phase can uniformly fill the interstices between periclase grains and surround coarse chromite particles [24]. This not only strengthens the matrix bonding but also effectively fills some open pores due to the volume expansion accompanying its formation.

The black areas in Figure 5 represent pores. Five pores in the main region of the image were selected for pore size measurement using ImageJ software, and the average value was 76.5 μm.

An excessive addition of Cr_2_O_3_ micro-powder, on the contrary, reduces the mechanical properties of the material. This is due to the thermal expansion coefficient mismatch between MgO (13.5 × 10^−6^/°C) and MgCr_2_O_4_ (8 × 10^−6^/°C), which generates excessive microcracks in the microstructure and leads to increased porosity [24]. The experimental results indicate that the optimal performance was achieved with 2 wt.% Cr_2_O_3_ micro-powder addition. Excessive addition of Cr_2_O_3_ micro-powder degrades the mechanical properties of the material. As shown in the microstructure (Points 58 and 61), the in situ-formed MgCr_2_O_4_ spinel is closely intergrown with the MgO matrix. The formation of spinel is accompanied by a volume expansion of approximately 7–8%, which, combined with the thermal expansion coefficient mismatch between MgO (13.5 × 10^−6^/°C) and MgCr_2_O_4_ (8 × 10^−6^/°C) [24], induces localized stress concentration at the phase boundaries. As clearly observed around these spinel–MgO interfaces, this stress leads to the generation of numerous microcracks. These micro-defects act as crack initiation sites under loading, thereby reducing the material’s strength and structural integrity. Consistent with this mechanism, the experimental results show that the optimal comprehensive performance is achieved at a Cr_2_O_3_ micro-powder addition of 2 wt.%.

#### 3.1.3. Study on the Addition Amount of Sintered Magnesia Powder

From the above research, it is known that the formulation with 2 wt.% Cr_2_O_3_ micro-powder and 3 wt.% chromite powder yields favorable sintering results. To further enhance the sintering performance, we conducted an investigation into the effect of sintered magnesia powder on the sintering properties of magnesia–chrome bricks, with the specific formulations detailed in Table 2. With the increase in sintered magnesia powder, the cold compressive strength of the non-impregnated samples increased from 32.65 MPa to 41.73 MPa, and the linear change rate decreased from 0.25% to 0.06%, exhibiting a clear sintering trend. The bulk density and apparent porosity showed little change, indicating a certain degree of densification within this proportion range and improved volume stability.

**Figure 7:**
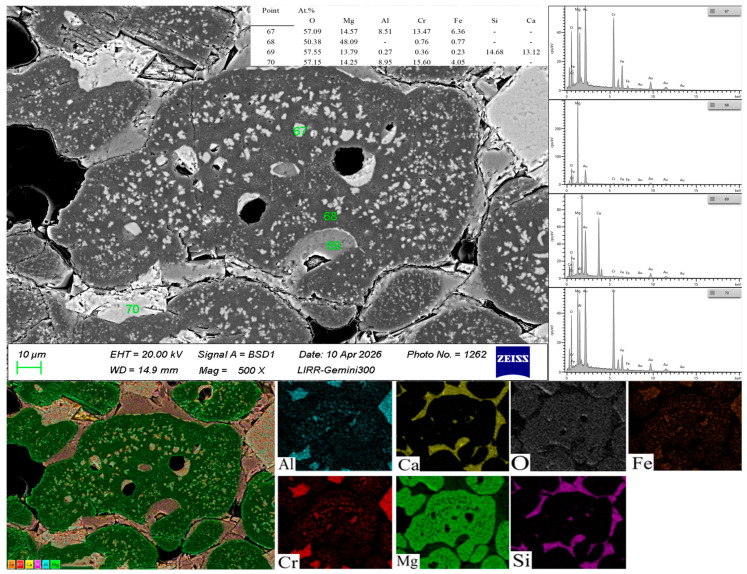
Microstructure image of the sample with 11 wt.% sintered magnesia powder.

As can be seen from Figure 7, a large number of bright-white spots are dispersed in the fine sintered magnesia powder and the white spots are identified as magnesia–chrome spinel by elemental analysis. Point 67 is magnesia–chrome spinel precipitated from the MgO matrix; point 68 is the magnesia matrix containing a very small amount of Cr element; point 70 is intergranular magnesia–chrome spinel formed by the reaction between Cr_2_O_3_ micro-powder and periclase. Sintered magnesia powder contains more lattice defects and exhibits a higher specific surface area than fused magnesia powder. Meanwhile, it has a slightly lower MgO content and contains moderate CaO and SiO_2_ impurities. These characteristics promote grain refinement of periclase and provide numerous active grain-boundary sites for Cr diffusion. As a result, the solid-state reaction is significantly accelerated, contributing to the sufficient and uniform formation of the spinel phase.

### 3.2. Analysis and Discussion of Salt Impregnation Treatment

MgSO_4_ exhibits high elemental compatibility with the magnesia–chrome brick matrix, along with advantages such as excellent solution permeability, low cost, and ease of industrial scalability. After magnesium salt impregnation treatment, the copper matte corrosion resistance and hydration resistance of magnesia–chrome refractory bricks are improved [25]. The magnesium salt filled in the pores decomposes at a certain temperature, leaving behind MgO, which is compatible with the matrix.

#### 3.2.1. Effect of Salt Immersion Treatment on Room-Temperature and High-Temperature Properties

**Figure 8:**
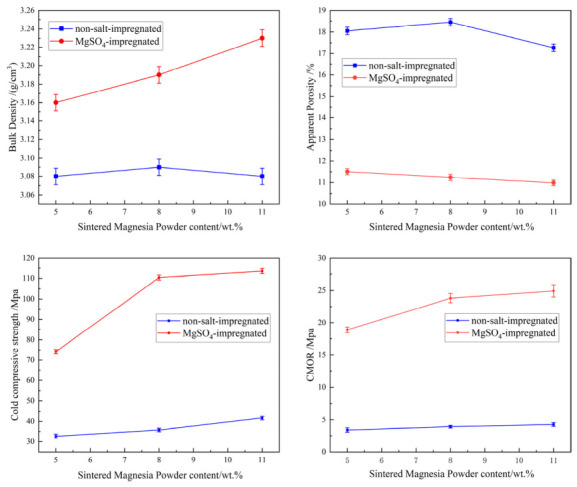
Effect of sintered magnesia powder content and salt impregnation on performance.

The data in Figure 8 show that after salt impregnation, the apparent porosity of the samples decreased from approximately 18% to the range of 10–12%, while the bulk density, cold compressive strength, and cold modulus of rupture increased significantly. This indicates that after the MgSO_4_ salt solution fills the open pores and cracks inside the brick, the material’s densification is greatly enhanced. Coupled with the certain chemical bonding effect of MgSO_4_, it effectively hinders crack propagation, thereby substantially increasing the material’s fracture energy, and consequently improving its cold compressive strength and modulus of rupture.

**Figure 9:**
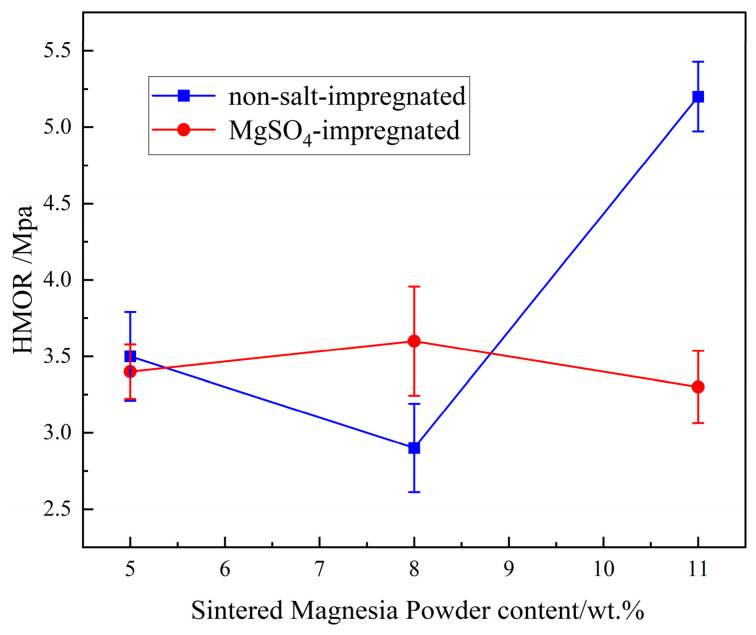
Effect of sintered magnesia powder content and salt impregnation on HMOR.

The effect of sintered magnesia powder on the hot modulus of rupture of the material before and after salt impregnation is shown in Figure 9. It can be seen from the figure that for the non-impregnated samples, as the sintered magnesia powder increased, the hot modulus of rupture first decreased from 3.5 MPa to 2.9 MPa and then increased to 5.2 MPa. For the samples after salt impregnation, it first increased from 3.4 MPa to 3.6 MPa and then decreased to 3.3 MPa. Following impregnation, the hot modulus of rupture (HMOR) of sample 11 decreased from 5.2 MPa to 3.3 MPa, while that of sample 10 increased from 2.9 MPa to 3.6 MPa. In contrast, sample 9 exhibited a slight reduction. Overall, the HMOR of the salt-impregnated samples did not show a consistent enhancement; instead, it displayed fluctuating decreases or instability across different formulations.

Salt impregnation treatment significantly improves the ambient-temperature mechanical properties of the material, but its effect on the high-temperature modulus of rupture is more complex. After drying at 110 °C, MgSO_4_ fills the pores in a crystalline state, increasing the material’s densification and decreasing its porosity. In the absence of a reducing agent, the decomposition temperature of MgSO_4_ is 1080 °C [26]. MgSO_4_ thermally decomposes to form MgO and SO_3_ (escape), without generating any low-melting-point impurity phases. The decomposition process is accompanied by the escape of SO_3_ gas, which induces the formation of micropores and microcracks at the original filling locations, preventing further improvement of the material’s high-temperature properties. However, since the residual MgO from the decomposition of the magnesium salt at high temperatures is compatible with the matrix [25,26] and does not form any harmful phases, the high-temperature performance of the material is comparable to, or even slightly better than, that of non-impregnated samples. This process provides a stable and feasible technical route for the low-cost industrial preparation of high-performance magnesia–chrome bricks.

#### 3.2.2. Microstructural Analysis After Salt Impregnation

**Figure 10:**
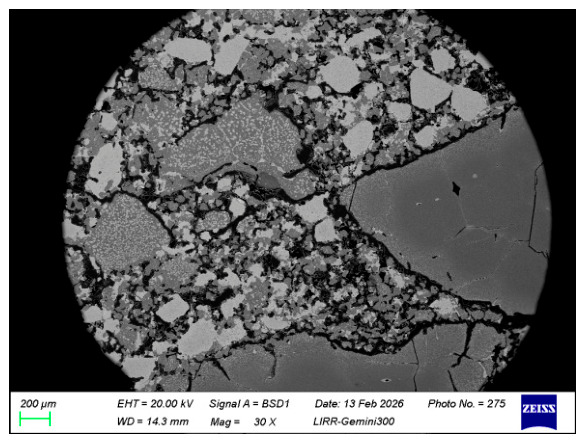
SEM image of salt-impregnated magnesia-chrome brick.

**Figure 11:**
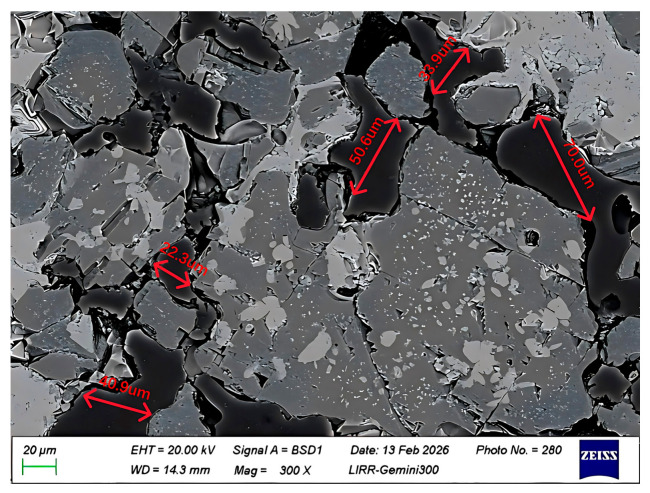
Microstructure of matrix phase in salt-impregnated magnesia-chrome brick.

Figure 10 shows the microstructure of the sample after salt impregnation. It can be seen from the figure that the material structure is relatively dense. The white particles are chromite and the two large particles at the bottom are fused magnesia. A certain amount of chromic oxide micro-powder has been incorporated into the edges of the fused magnesia, forming a magnesia–chrome spinel mineral phase. Figure 11 shows the microstructure of the matrix of the sample after salt impregnation. The fine powder distribution in the matrix is relatively uniform. The added chromic oxide micro-powder has been incorporated into the magnesia fine powder, and the white areas formed at the matrix bonding sites are all magnesia–chrome spinel mineral phases. The chromite fine powder, due to its higher impurity content, is more prone to sintering together with other fine powders.

**Figure 12:**
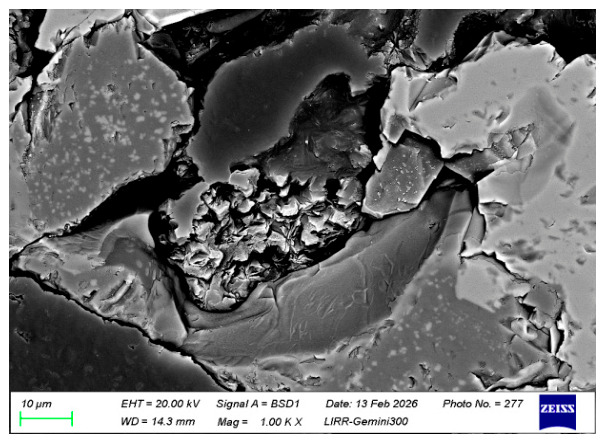
Pore filling morphology in salt-impregnated magnesia-chrome brick (low magnification).

**Figure 13:**
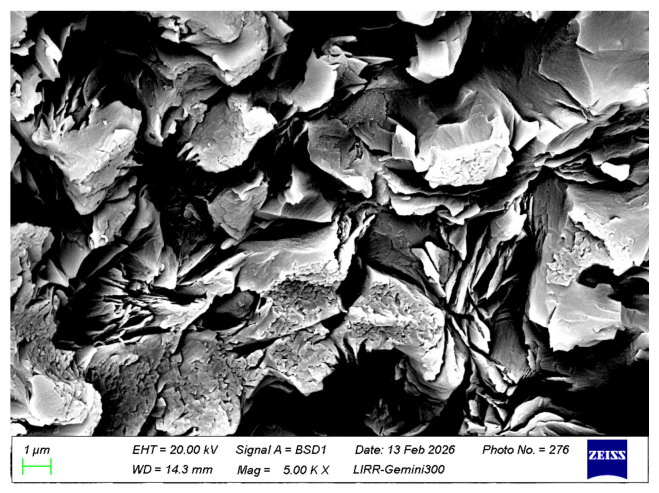
Micro-Pillar reinforcement structure in pores of salt-impregnated magnesia-chrome brick (high magnification).

Figure 12 and Figure 13 are microstructural images of the pore regions in the sample after salt impregnation, clearly showing that after MgSO_4_ salt impregnation treatment, the salt solution crystallized within the pores, forming a unique stamen-like microstructure. The crystalline salt, appearing as interwoven needle-like and rod-like shapes, uniformly fills the interior of the open pores, creating a three-dimensional micro-pillar reinforcing phase. This structure not only fills the open pores of the material, significantly reducing the apparent porosity, but also effectively hinders crack initiation and propagation under load by dispersing stress concentration. This is the core microscopic reason for the significant improvement in the material’s ambient-temperature mechanical properties after salt impregnation. The average pore size of the main region in Figure 12 is 43.54 μm. After salt impregnation, the apparent porosity of the material decreases, and the pore size becomes smaller and more uniform. Meanwhile, the stamen-like crystalline salt is tightly bonded to the pore walls without obvious interfacial gaps, indicating that the MgSO_4_ salt solution fully penetrated into the interior of the pores under vacuum pressure, achieving pore filling.

**Figure 14:**
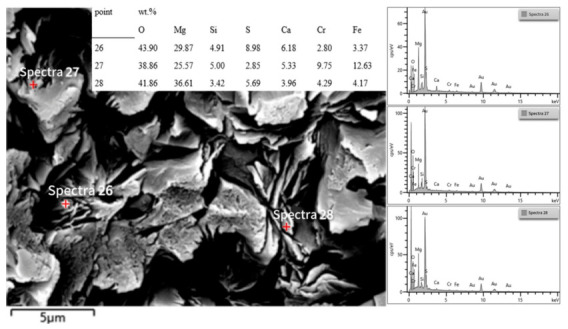
EDS spectrum analysis diagram of the sample matrix after salt impregnation.

According to the EDS spectrum analysis in Figure 14 and the oxide content detection in Table 6, spectrum 26, taken from the pore-filling region, detected high contents of Mg, S, and O elements, along with small amounts of Si, Ca, Cr, and Fe. This confirms that the filling phase is primarily MgSO_4_ crystalline salt, intermixed with a small amount of matrix components. The interfacial fusion between the salt solution and the matrix enhances the bonding force between the filling phase and the matrix. Spectra 27 and 28 were taken from different regions of the matrix. The results show relatively high contents of elements such as Cr, Fe, Mg, and Si, corresponding to the magnesia–chrome spinel phase and a small amount of silicate phase. Specifically, in Spectrum 27, the Cr_2_O_3_ content reached 14.24% and the Fe_2_O_3_ content reached 18.06%, confirming that this region is rich in chromite and its reaction products. In Spectrum 28, the MgO content reached 60.69%, corresponding to a periclase-enriched area. Additionally, a small amount of SO_3_ (14.22%) was detected, indicating that under the action of vacuum pressure, some of the MgSO_4_ salt solution not only filled large-sized open pores but also penetrated into the micron-sized micropores of the matrix, further enhancing the overall densification of the material.

Comprehensive analysis reveals that after firing the optimized formulation at 1750 °C, a uniformly distributed secondary magnesia–chrome spinel phase formed within the matrix, achieving direct bonding between particles. The vacuum-pressure salt impregnation treatment allowed the MgSO_4_ molten salt to fully fill the open pores, forming a stamen-like crystalline reinforcing structure. The synergistic effect of these two factors endowed the material with a dense microstructure, providing the structural foundation for its excellent mechanical properties. However, the EDS results simultaneously showed residual sulfur elements in some areas after salt impregnation. At high temperatures, these residues may decompose or form low-melting-point phases, providing a microscopic basis for explaining the fluctuation in the high-temperature modulus of rupture after salt impregnation.

### 3.3. Performance Comparison with RHI Products

To better analyze the feasibility and pioneering nature of the preparation method presented in this paper, a performance comparison was conducted between the optimal formulation from this study (11 after salt impregnation) and a high-end magnesia–chrome brick products from RHI Magnesita (Austria), one of the most renowned international brands of refractory products. The product data of RHI Magnesita(as shown in Table 7) were obtained by testing officially purchased products. All performance tests in this study strictly followed Chinese national standards. This comparison is used to benchmark the performance range and industrial level, and to determine whether the samples from this study reach the performance range of international high-end magnesia–chrome bricks.

The results show that the bulk density (3.23 g/cm^3^) and apparent porosity (10.98%) of the 11 sample after salt impregnation have reached the level of the RHI Magnesita product; the cold compressive strength (113.52 MPa) is even superior to that of the comparable RHI Magnesita product; and the linear change rate (0.06%) demonstrates even better volume stability, providing a guarantee for structural integrity during high-temperature service.

## 4. Conclusions and Future Research Directions

This study systematically investigated the effects of component composition optimization and vacuum-pressure salt impregnation treatment on the properties of magnesia–chrome refractory, while also examining the sintering performance and underlying mechanisms of high-performance magnesia–chrome bricks. It successfully produced a material whose key performance indicators are comparable to, or even better than, those of imported high-end products. The main conclusions are as follows:

(1) The optimal formulation was determined to be 3 wt.% chromite powder and 2 wt.% Cr_2_O_3_ micro-powder, which enables the in situ formation of uniform magnesia–chrome spinel, constructing a spinel bridge network and significantly enhancing the matrix bonding strength.

(2) Sintered magnesia powder enhances material densification and volume stability through impurity-assisted sintering and grain refinement effects. With an addition of 11 wt.%, the linear change rate is as low as 0.06%.

(3) The MgSO_4_ vacuum salt impregnation process leads to the formation of a micro-pillar reinforcement structure via crystalline salt filling, resulting in a cold compressive strength exceeding 110 MPa, reaching the level of international high-end magnesia–chrome bricks.

(4) At high temperatures, MgSO_4_ decomposes to produce MgO and SO_3_, causing fluctuations in the high-temperature modulus of rupture. However, the residual MgO is compatible with the matrix, and the material’s high-temperature performance shows no significant degradation.

Based on the above results and discussion, we will further conduct in-depth research on the following aspects: (1) To establish a quantitative relationship between spinel content, spinel type, and mechanical strength of magnesia–chrome refractories. (2) To systematically evaluate the slag corrosion resistance and thermal shock resistance of the salt-impregnated samples.

## Figures and Tables

**Table 1:** Main component contents of raw materials.

Raw Materials	wt.%					
	Cr_2_O_3_	Al_2_O_3_	MgO	CaO	SiO_2_	Fe_2_O_3_
Chromite Powder	47.68	14.27	10.08	0.21	0.82	25.53
Fused Magnesia–Chrome	22.32	-	64.28	1.10	1.16	9.58
Fused Magnesia Powder	-	0.14	98.11	1.03	0.25	0.41
Sintered Magnesia Powder	-	0.17	97.06	1.42	0.59	0.37

**Table 2:** Formulation (wt.%).

Formulation	1	2	3	4	5	6	7	8	9	10	11
Fused Magnesia	30%	30%	30%	30%	30%	30%	30%	30%	30%	30%	30%
Fused Magnesia–Chrome	26%	26%	26%	26%	26%	26%	26%	26%	26%	26%	26%
Chromite	10%	10%	10%	10%	10%	10%	10%	10%	10%	10%	10%
Fused Magnesia Powder	33%	30%	27%	21%	30%	29%	28%	27%	23%	20%	17%
Sintered Magnesia Powder	0%	0%	0%	0%	0%	0%	0%	0%	5%	8%	11%
Cr_2_O_3_ Micro-Powder	0%	0%	0%	0%	0%	1%	2%	3%	2%	2%	2%
Chromite Powder	0%	3%	6%	12%	3%	3%	3%	3%	3%	3%	3%
Pulp Powder	1%	1%	1%	1%	1%	1%	1%	1%	1%	1%	1%

**Table 3:** Sample performance.

Formulation	Bulk Density /(g/cm^3^)	Apparent Porosity/%	Cold Compressive Strength/Mpa	CMOR /Mpa
1	3.09	19.72	19.53	2.52
2	3.10	18.23	20.22	3.56
3	3.07	19.86	18.98	2.49
4	3.02	19.99	17.54	2.27
5	3.10	18.23	20.22	3.56
6	3.05	18.16	20.62	3.60
7	3.09	17.82	25.12	3.82
8	3.08	18.12	23.36	3.71

**Table 4:** Performance of non-salt-impregnated specimens.

Formulation	Linear Change Rate/%	Bulk Density /(g/cm^3^)	Apparent Porosity /%	Cold Compressive Strength/Mpa	CMOR/Mpa	HMOR /Mpa
9	0.25	3.08	18.05	32.65	3.39	3.5
10	0.32	3.09	18.45	35.76	3.92	2.9
11	0.06	3.08	17.27	41.73	4.25	5.2

**Table 5:** Performance of MgSO_4_-impregnated specimens.

Formulation	Linear Change Rate/%	Bulk Density /(g/cm^3^)	Apparent Porosity /%	Cold Compressive Strength/Mpa	CMOR/Mpa	HMOR /Mpa
9	0.25	3.16	11.51	74.17	18.88	3.4
10	0.32	3.19	11.23	110.42	23.82	3.6
11	0.06	3.23	10.98	113.52	24.91	3.3

**Table 6:** Oxide content at the test point.

Point wt.%						
	MgO	SiO_2_	SO_3_	CaO	Cr_2_O_3_	Fe_2_O_3_
26	49.52	10.50	22.42	8.65	4.09	4.82
27	42.40	10.70	7.13	7.46	14.24	18.06
28	60.69	7.31	14.22	5.54	6.27	5.96

**Table 7:** Performance data of magnesia–chrome refractory bricks from RHI Magnesita (Austria).

Brand	Bulk Density /(g/cm^3^)	Apparent Porosity/%	Cold Compressive Strength/Mpa
Radex-ZP80	2.90–3.05	17–21	35–70
Radex-DB3805	3.00–3.15	15–19	30–70
Radex-DB70	2.90–3.05	17–21	30–60
Radex-BC(4.6)	3.10–3.30	11–18	40–100
Radex-H60	3.05–3.25	16–20	35–70
Radex-DB505	3.15–3.30	15–19	35
Radex-BCF33	3.25–3.40	14–17	50–100

## Data Availability

The original contributions presented in this study are included in the article. Further inquiries can be directed to the corresponding author.
